# Regulation of the Gα-cAMP/PKA signaling pathway in cellulose utilization of *Chaetomium globosum*

**DOI:** 10.1186/s12934-018-1008-6

**Published:** 2018-10-11

**Authors:** Yang Hu, Yanjie Liu, Xiaoran Hao, Dan Wang, Oren Akhberdi, Biyun Xiang, Xudong Zhu

**Affiliations:** 1 Department of Pathogen Biology, School of Basic Medical Sciences, Tianjin Medical University, Tianjin, China; 2 Beijing Key Laboratory of Genetic Engineering Drug and Biotechnology, Institute of Biochemistry and Biotechnology, School of Life Sciences, Beijing Normal University, No. 19, XinJieKouWai St., HaiDian District, Beijing, 100875 China; 3 National Key Program of Microbiology and Department of Microbiology, College of Life Sciences, Nankai University (DMNU), Tianjin, China

**Keywords:** *Chaetomium globosum*, Heterotrimeric GTP binding protein, cAMP, PKA, Cellulase, Xylanase

## Abstract

**Background:**

The canonical heterotrimeric G protein-cAMP/PKA pathway regulates numerous cellular processes in filamentous fungi. *Chaetomium globosum*, a saprophytic fungus, is known for producing many secondary metabolites, including cytotoxic chaetoglobosin A (ChA), as well as abundant cellulase and xylanase.

**Results:**

Here we report on the functional characterization of this signaling pathway in *C. globosum*. We blocked the pathway by knocking down the putative Gα-encoding gene *gna1* (in the pG14 mutant). This led to impaired cellulase production and significantly decreased transcription of the major cellulase and xylanase genes. Almost all the glycohydrolase family genes involved in cellulose degradation were downregulated, including the major cellulase genes, *cel7a*, *cel6a*, *egl1*, and *egl2.* Importantly, the expression of transcription factors was also found to be regulated by *gna1*, especially Ace1, Clr1/2 and Hap2/3/5 complex. Additionally, carbon metabolic processes including the starch and sucrose metabolism pathway were substantially diminished, as evidenced by RNA-Seq profiling and quantitative reverse transcription (qRT)-PCR. Interestingly, these defects could be restored by simultaneous knockdown of the *pkaR* gene encoding the regulatory subunit of cAMP-dependent PKA (in the pGP6 mutant) or supplement of the cAMP analog, 8-Br-cAMP. Moreover, the Gα-cAMP/PKA pathway regulating cellulase production is modulated by environmental signals including carbon sources and light, in which VelB/VeA/LaeA complex and ENVOY probably work as downstream effectors.

**Conclusion:**

These results revealed, for the first time, the positive role of the heterotrimeric Gα-cAMP/PKA pathway in the regulation of cellulase and xylanase utilization in *C. globosum.*

**Electronic supplementary material:**

The online version of this article (10.1186/s12934-018-1008-6) contains supplementary material, which is available to authorized users.

## Background

Cellulose, together with hemicellulose and lignin, is a major component of lignocellulosic biomass. Because it is difficult to degrade, it remains in the natural environment as waste [1]. In recent years, the worsening energy crisis and environmental pollution have shifted the focus of energy generation to the reuse of cellulolytic waste [2]. Biological degradation of lignocellulose into fermentable sugars by cellulosic enzymes is a promising and environmental-friendly approach. The degradation is performed by three classes of enzymes: endoglucanases (EGs) (EC 3.2.1.4), exo-glucanases or cellobiohydrolases (CBHs) (EC 3.2.1.91), and β-glucosidases (BGLs) (EC 3.2.1.21). Moreover, cellobiose dehydrogenase (CDH) (EC 1.1.99.18) acts synergistically with canonical glycohydrolases (GHs), accelerating the enzymatic conversion of polysaccharides [2–4]. In addition, there are many endo- and exo-acting enzymes that attack the heterogeneous hemicelluloses, such as endo-β-1,4-xylanase, β-xylosidase, and mannanase [5].

The exploration and screening of fungal strains that can use cellulose provides a potential approach to improving the reuse of cellulolytic waste [6]. Some ascomycetes such as *Trichoderma reesei* and *Aspergillus nidulans* are commonly used for producing cellulases and hemicellulases in industry [7, 8]. In addition, *Chaetomium globosum* is a saprophytic fungus with a high capability for degrading plant materials, such as wheat straw, coffee pulp, and oil palm empty fruit bunch (OPEFB) fiber to produce cellulase [9]. Recently, the cellulose- degrading enzyme system of *C. globosum* has been studied, and the results suggest an excellent potential for developing a cellulase-producing strain for on-site enzyme production [10]. However, the transcriptional regulation mechanism of cellulase production in *C. globosum* is still not clear. In the past few decades, transcriptional regulation of cellulases has been extensively studied in some fungi [11]. Several important transcription factors (TFs) have been found to be involved in these processes [12], with many related regulatory mechanisms being conserved in different fungal species [13, 14]. For example, some TFs, including CreA, Ace1, Ace2 [15], XlnR [16, 17], Hap2/3/5 complex [13], and Clr1/2 [18], have been found to regulate the expression of cellulases in filamentous fungi. Apart from Ace2, orthologs of the other transcription regulators were found in *C. globosum* [19]. CreA in *Aspergillus* sp. and Cre1 in *Hypocrea jecorina* act as carbon catabolite repressors, modulating cellulase gene transcription [15]. Ace1 acts as a transcriptional repressor in *H. jecorina*, while it acts as an activator in *Saccharomyces cerevisiae* [20]. In addition, the Hap2/3/5 complex, which binds to the cis element (known as the “CCAAT motif”), positively regulates many cellulases and hemicellulases [21]. Unfortunately, research on how the polysaccharide signals are transmitted to the TFs is very rare [22]. A preliminary analysis of putative homologs of the TFs and the distribution of consensus sequences suggests that *C. globosum* may have a peculiar regulatory mechanism for genes involved in cellulose degradation [19]. Nevertheless, the mechanism of cellulase production in *C. globosum* and the pathways that transduce signals to specific TFs are thus far unclear.

To improve the efficiency of hydrolytic enzyme production in filamentous fungi, except for the transcriptional regulation mechanisms, it is also indispensable to study signaling pathways controlling protein synthesis and secretion. Signal transduction via heterotrimeric G proteins has been studied in numerous fungi and is now recognized as one of the most important types of signaling pathway regulating growth and conidiation, sexual development, virulence, tolerance of various forms of stress, and secondary metabolite production [23]. In filamentous fungi, the sensing of depleted carbon sources or amino acids by a G protein-coupled receptor (GPCR) activates the coupled Gα subunit of the G protein complex that in turn transfers the signal to adenylyl cyclase, to regulate the in vivo cyclic adenosine monophosphate (cAMP) levels. cAMP produced by adenylyl cyclase can bind to the regulatory subunit of cAMP-dependent PKA (PKA-R), leading to activation of the PKA catalytic subunit (PKA-C) and phosphorylation of downstream targets in the pathways associated with fundamental biological functions in response to extracellular signals [24–27]. It has been suggested that induction of cellulases is related to a signaling pathway involving a cAMP-dependent protein kinase in several fungi, for example, *T. reesei* [8, 28], *A. nidulans* [7], and *Penicillium decumbens* [29]. However, the role of the G protein-cAMP/PKA signaling pathway in the transcriptional regulation of cellulases in *C. globosum* has not been defined.

As part of our ongoing effort into understanding the regulation of cellulase production in *C. globosum*, we started with defining the role of the G protein-cAMP/PKA pathway in the transcriptional regulation of cellulases. The fungal strain *C. globosum* NK102 was formerly isolated as a high-yield chaetoglobosin A (ChA) producer that could use cellulose as a sole carbon source [30–32]. To gain insight into the regulatory mechanism of ChA biosynthesis in *C. globosum*, in the previous study, we knocked down the glucosamine 6-phosphate *N*-acetyltransferase 1 (*gna1*) gene (homolog of CHGG_03321 of *C. globosum* CBS 148.51), which putatively encodes a group I Gα protein, and the gene *pkaR* (equivalent of CHGG_00688 of *C. globosum* CBS 148.51), which encodes the regulatory subunit of the cAMP-dependent PKA, using an established RNA interference (RNAi) strategy [30, 33]. The results showed that the canonical G protein-cAMP/PKA pathway plays a pivotal role in the production of ChA in *C. globosum* NK102. We generated a knockdown mutant of the gene *gna1* (the pG14 mutant) and a knockdown mutant of both *gna1* and *pkaR* (the pGP6 mutant) [34]. In this study, we further demonstrated that the transcriptional regulation of cellulase genes involves the G protein-cAMP/PKA pathway. The regulation was linked to the intracellular cAMP level and the expression of associated TFs and other regulators. In addition, this signaling pathway is modulated by environmental factors including carbon sources and light conditions.

## Methods

### Fungal strains and culture conditions

The strains used in this study included the wild-type *C. globosum* strain NK102, pG14 (a *gna1*-silenced transformant), and pGP6 (a *gna1*- and *pkaR*-double silenced transformant). The wild-type strain *C. globosum* NK102, isolated and stocked at our laboratory, was used as the host strain for the RNAi experiments [30]. The pG14 and pGP6 transformants used in this work were obtained by RNAi. pG14 was obtained by transforming the protoplasts of *C. globosum* NK102 with the RNAi cassette pGNA-1, which contains two inverted complementary fragments of the 5′ end of *gna1.* pGP6 was obtained by transforming the protoplasts of *C. globosum* NK102 with the RNAi cassette pGNA-PKAR, which can simultaneously knockdown *gna1* and *pkaR*. Southern blot results proved that the RNAi cassettes were inserted into all the transformants [34]. Quantitative reverse transcription (qRT)-PCR results verified that *gna1* was knocked down in pG14, and *gna1* and *pkaR* were simultaneously knocked down in pGP6 [34].

The culture conditions of the wild-type and mutant strains have been described in previous work [34]. For DNA or RNA isolation, 5-mm agar plaques containing the fungal hypha inoculated in potato dextrose broth were incubated for 4 or 8 days in a rotary shaker at 28 °C and 180 rpm. For RNA-Seq analysis, 5-mm agar plaques containing the hypha of the wild-type strain or transformants were inoculated in microcrystalline cellulose (MCC) medium (per liter: 10 g microcrystalline cellulose, 1.4 g (NH4)_2_SO_4_, 2.0 g KH_2_PO_4_, 0.3 g urea, 0.3 g MgSO_4_·7H_2_O, 0.3 g CaCl_2_, 1.0 g peptone, and trace elements, i.e., 5 mg FeSO_4_·7H_2_O, 1.56 mg MnSO_4_·H_2_O, 1.67 mg ZnCl_2_, and 2.0 mg CoCl_2_) and incubated for 8 days, shaking at 28 °C and 180 rpm. Strains were grown in constant illumination (2000 lx) in constant temperature incubator shake LRH-250-Z (Guangdong, China). For gene expression assays and cellulase activity measurements, a suspension containing approximately 10^8^ spores/ml was inoculated in 200 ml MCC medium [19] with 1% (w/v) lactose or 1% (w/v) glucose as a carbon source, and with or without 2 mM of the PKA activator 8-bromoadenosine-3′,5′-cyclic monophosphate (8-Br-cAMP) (Sigma–Aldrich, USA), which is an analog of cAMP.

### RNA-Seq, data mining and gene ontology analysis

RNA-Seq profiling was carried out by a commercial provider to monitor the consequences associated with the knockdown mutants. Illumina HiSeq™ sequencing of total mRNA from the wild-type strain or the transformant pG14 was conducted by BGI (Shenzhen, China; http://en.genomics.cn/navigation/index.action). The genome sequence of wild-type *C. globosum* NK102, has high level homology with *C. globosum* CBS 148.51, was used as the reference for the analysis (unpublished data). P-values were used to evaluate the statistical significance of expression differences [35]. A false discovery rate (FDR)-corrected P-value < 0.001 and an absolute log_2_Ratio value ≥ 1 were used to identify the differentially expressed genes (DEGs) and differentially expressed tags (DETs).

### RNA isolation and qRT-PCR

Total RNA was extracted from the lyophilized and ground mycelium using a TRIzol kit (Invitrogen, CA, USA), and it was then treated with RNase-free DNase (Takara Inc, Dalian, China) to remove possible contaminant DNA. The first-strand cDNA was generated by reverse transcription in a 20-μl reaction using a Moloney Murine Leukemia Virus (M-MLV) RTase cDNA synthesis kit (Takara Inc.). qRT-PCR was performed on a Mastercycler PCR system (Eppendorf, Germany) using SYBR green as a fluorescence reporter (BioRad, CA, USA) following the manufacturer’s protocol. Reactions were set up based on three replicates per sample. Controls without the addition of the templates were included for each primer set. The PCR cycling parameters were as follows: pre-incubation at 94 °C for 10 min, followed by 40 cycles of denaturation at 94 °C for 15 s, annealing at 59 °C for 30 s, and extension at 72 °C for 32 s. The expression of each gene of interest (expressed as the Ct value) was normalized against β-actin mRNA. The method has been described previously [30]. The qRT-PCR data were analyzed using the 2^−ΔΔCt^ relative quantification method [36]. The primers for RT and PCR are provided in Additional file 1: Table S1.

### Cellulose degradation experiment and enzyme assays

Screening for cellulase producers was carried out on carboxymethylcellulose (CMC) agar (0.2% NaNO_3_, 0.1% K_2_HPO_4_, 0.05% MgSO_4_, 0.05% KCl, 0.2% CMC sodium salt, 0.02% peptone, and 1.7% agar). After incubation at 28 °C for 4 days, the plates were flooded with Gram’s iodine (2.0 g KI and 1.0 g iodine in 300 ml distilled water) for 5 min [37]. The radius of the zone of clearance around each colony observed was measured to compare the cellulose-utilizing ability between strains.

For the cellulase activity assays, strains were cultivated in 500-ml Erlenmeyer flasks containing 200 ml MCC medium, with constant shaking at 120 rpm and 28 °C. After 8 days of incubation, the fermentation broth was used as the enzyme source. Filter paper assays for assessing saccharifying cellulase activity and carboxymethyl cellulase (CMCase) assays for assessing endo-3-l,4-glucanase activity were carried out according to methods designed by Eveleigh et al. [38]. Xylanase assays for endo-1,4-β-xylanase were performed using Azo-Xylan (Birchwood, USA) as the substrate, according to the manufacturer’s instructions. BGL activity was measured using 20 μl culture supernatant. 4-Nitrophenyl beta-d-glucopyranoside (PNP-Glu) in 50 mM buffer citrate (pH 6.0) was used as the substrate for the BGL activity assay, as previously described [39]. All enzyme assays were carried out on the supernatants of biological triplicates. Biological experiments were independently performed in triplicate.

## Results and discussion

### Knockdown of *gna1* leads to defects in cellulose-utilising ability and the defects can be reversed by simultaneous knockdown of *gna1* and *pkaR*

**Fig. 1:**
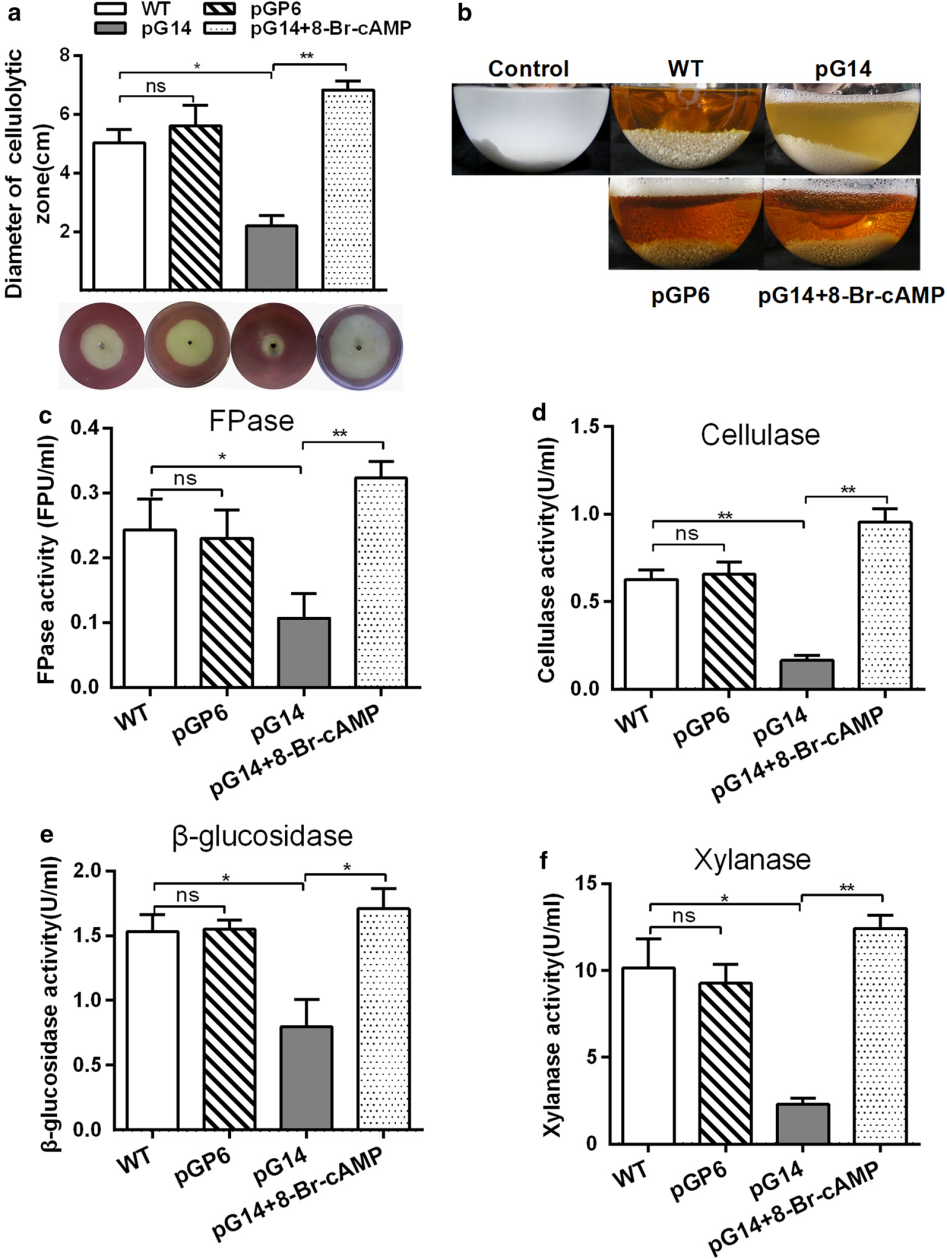
Knockdown of *gna1* results in a decrease in secreted hydrolytic enzymes. **a** Radius of the zone of hydrolysis in carboxymethylcellulose (CMC) medium; **b** phenotype of strains in microcrystalline cellulose (MCC) medium; **c** filter-paper-hydrolyzing (FPase) activity; **d** carboxymethylcellulose (CMCase) activity; **e** β-glucosidase (BGL) activities; **f** xylanase activities in different strains with or without 2 mM 8-Br-cAMP. Mycelia were grown in 500-ml Erlenmeyer flasks containing 200 ml MCC medium, with constant shaking at 120 rpm and 28 °C. After 8 days of incubation, the fermentation broth was used as the enzyme source. The statistical significance is indicated by an asterisk (P-value < 0.05 in t-test analysis) or by two asterisks (P-value < 0.01 in t-test analysis). Experiments were carried out in biological triplicates

To investigate whether the G protein signaling pathway was involved in the production of cellulase, the cellulose-utilizing ability and cellulase activity of the pG14 and pGP6 mutants and the wild-type strain were detected. After incubation on CMC agar for 4 days, the plates of all the strains were flooded with Gram’s iodine for 5 min [37]. By comparing the diameter of the zone of clearance around each colony observed, we found that the diameter of pG14 was dramatically smaller than that of the wild-type strain, which means that the cellulose-utilizing ability of the mutant decreased (Fig. 1a). The diminished diameter was restored to approximately the wild-type level in pGP6. Furthermore, the MCC liquid medium became clear after inoculation of the wild-type strain at 28 °C for 8 days, while pG14 had a turbid (rather than clear) phenotype, indicating that its cellulose-utilizing ability was decreased (Fig. 1b). When the wild type strain was cultured in MCC liquid medium for 4 days and 8 days respectively, we observed that cellulase activity and the cellulase gene expression increased with time in the fungal cultures (Additional file 1: Table S2). Therefore, further studies on cellulase activity and the cellulase gene expression were all cultured for 8 days. Filter-paper-hydrolyzing (FPase) activity, CMCase activity, xylanase activity, and BGL activity of the wild-type strain and mutants were also detected (Fig. 1c–f). In 8-day-old fermentation broths, cellulase activity level was significantly lower in the pG14 mutant compared to in the wild-type strain, yet the activity level recovered to some extent in pGP6 compared to the initial deficient phenotype of pG14. For instance, the cellulase and xylanase activity levels of pG14 compared to the wild-type strain decreased to 26.1% and 22.7%, respectively. As shown in Fig. 1, the diminished level of cellulase and xylanase activity was restored to approximately the wild-type level in pGP6, at 104.8% and 91.4%, respectively. Hence, *gna1* is clearly critical for the cellulose-utilizing ability in *C. globosum* NK102. The phenotype showed by the double knockdown mutant suggests that PkaR is the downstream effector of Gna-1 regarding cellulase biosynthesis. More specifically, Gna-1 inhibits PkaR function, which negatively regulates the production of cellulase. This observation concurs with previous findings that the G protein-mediated signaling pathway is critical for cellulolytic enzyme secretion in other filamentous fungi [8, 29].

### Genome-wide profiling of gene expression in the *gna1* knock-down mutant pG14 using an RNA-Seq approach

A previous global gene expression analysis involving RNA-Seq enabled the construction of diagrams of gene regulatory networks that enhanced understanding of the interaction between different genes involved in cellulose degradation and metabolism [7]. To assess the global expression of genes that might be regulated by *gna1*, an RNA-Seq profiling analysis was performed to identify the DEGs in the *gna1* mutant pG14. DEGs between the wild-type strain and pG14 were selected based on FDR < 0.001 and |log_2_Ratio| ≥ 1. In pG14, among a total of 3326 DEGs, 688 were upregulated and 2638 were downregulated [34]. The result of the transcriptome analysis done by RNA-seq as a complementary data in our previous work is online (10.1371/journal.pone.0195553.s004).

The GO analysis of DEGs showed that pG14-specfic transcriptional alterations involved genes encoding proteins involved in proteolysis, protein metabolic process, cell cycle, tRNA biosynthesis and transport, secondary metabolite biosynthesis, cellular ketone metabolic process, etc. (Additional file 1: Table S3). The categories of genes that were specifically regulated in pG14 included proteins involved in the cellular protein metabolic process, cell cycle, protein transport and localization, and carbon metabolic process (Additional file 1: Table S3). This indicates that silencing of *gna1* resulted in the abnormal expression of genes involved in degradation pathways and a shift in metabolism to the use of carbon sources.

Interestingly, in the pathway enrichment analysis of pG14, in the starch and sucrose metabolism pathway, there were 46 downregulated genes and only 2 upregulated genes in pG14 (Additional file 1: Figure S1). According to the RNA-Seq results, almost all the GH family genes involved in the degradation of cellulose were downregulated in pG14 (approximately 2- to 28-fold), including the major cellulase genes, e.g., *cel7a* (CHGG_08475 and CHGG_08330), *cel6a* (CHGG_10762 and CHGG_06834), *egl1* (CHGG_10708 and CHGG_08509), and *egl2* (CHGG_01188) (Table 1). The genes with the highest coefficient of variation were CHGG_03421 (EC 3.2.1.37, β-xylosidase) and CHGG_07451 (EC 3.2.1.4, endoglucanase), the expression of which decreased to only 3.6% and 9.5%, respectively, compared to the expression in the wild-type strain. CHGG_10708 (*egl1*), which encodes an endoglucanase (EC 3.2.1.4) for attacking regions of low crystallinity in cellulose fibers, creating free chain-ends, displayed an expression of only 21.8% in pG14 compared to in the wild-type strain. The expression of CHGG_08330 (*cel7a*-*2*), the major cellobiohydrolase gene, dropped to 46.7% in pG14. In addition, the expression of CHGG_00030 and CHGG_00304 (*xln* genes) encoding endo-β-1,4-xylanase (EC 3.2.1.8), which attacks the heterogeneous hemicelluloses, decreased to 53.6% and 50%, respectively (Table 1). As expected, the transcription level of TFs also changed. According to the RNA-Seq results, the expression of *ace1* was significantly upregulated (log_2_Ratio = 0.5), while the expression levels associated with the *hap2/3/5* complex and *clr1/2* were down-regulated significantly (log_2_Ratio = − 1.8, − 3.2, − 2.4) (Table 1). The LaeA/VeA/VeB complex genes, which were previously reported to be involved in ChA biosynthesis and cellulase production [40], were down-regulated in pG14 as well (log_2_Ratio = − 1.7, − 0.5, − 1.7). In addition, the photoreceptor *env1* gene, connecting the light signaling and the cellulase gene transcript levels [41], was upregulated in pG14 in light (log_2_Ratio = 1.5) (Table 1).

**Table 1:** Variation of cellulase genes detected by RNA-seq profiling (RPKM > 20)

Gene number in *C. globosum* NK102	GH family/regulators	Protein/gene name predicted by Longoni et al. [19]	*C. globosum* gene ID (NCBI)	log_2_Ratio (WT/pG14)	FDR	Up/down-regulation (pG14/WT)
1	GH1	EC 3.2.1.21 β-glucosidase	CHGG_05642	− 1.3	2.32E − 23	Down
15	GH3	EC 3.2.1.37 β-xylosidase	CHGG_06807	− 1.8	2.56E−47	Down
			CHGG_01059	− 1.7	5.34E−79	Down
			CHGG_03421	− 4.8	0	Down
8	GH5	EC 3.2.1.4 endoglucanase/EC 3.2.1.78 mannase/*egl2*	CHGG_01188	− 0.6	0.000149	Down
1	GH45	EC 3.2.1.4 endoglucanase*/egl1*	CHGG_10708	− 2.2	7.68E−20	Down
			CHGG_08509	− 0.4	0.231624	Down
7	GH6	EC 3.2.1.4 endoglucanase*/*EC 3.2.1.91 cellobiohydrolase/*cel6a*	CHGG_10762	− 2.3	1.41E−09	Down
			CHGG_06834	− 1.0	0.002945	Down
8	GH7	EC 3.2.1.4 endoglucanase*/*EC 3.2.1.91 exoglucanase/*cel7a*	CHGG_08330	− 1.1	0.003731	Down
			CHGG_08475	− 0.5	0.011536	Down
30	GH61	EC 3.2.1.4 endoglucanase/copper-dependent polysaccharide monooxygenases	CHGG_03415	− 2.7	2.13E−09	Down
			CHGG_06059	− 1.2	8.34E−10	Down
			CHGG_08275	− 2.3	3.67E−31	Down
			CHGG_04473	− 0.5	0.078539	Down
			CHGG_00362	− 1.9	9.65E−09	Down
			CHGG_07756	− 1.8	1.02E−16	Down
			CHGG_07451	− 3.4	1.60E−23	Down
			CHGG_00683	− 1.4	1.01E−07	Down
			CHGG_07676	− 2.1	4.92E−05	Down
			CHGG_06144	− 2.1	4.92E−05	Down
6	GH10	EC 3.2.1.8 endo-1,4-xylanase/*xln*	CHGG_00030	− 0.9	0.077566	Down
			CHGG_00304	− 1.0	0.034787	Down
11	CDH	EC 1.1.9978 cellobiose dehydrogenase	CHGG_03380	− 1.8	0	Down
Negative regulators	AceI	*ace1*	CHGG_00776	0.5	3.78E−11	Up
	CreA	*cre1*	CHGG_03907	< − 0.1	0.601103	–
Positive regulators	XlnR	*xyr1*	CHGG_03981	Not detected		
	AceII	*ace2*	–			
	Hap2	*hap2*	CHGG_05974	− 0.7	4.99E−10	Down
	Hap3	*hap3*	CHGG_01529	− 1.8	1.57E−13	Down
	Hap5	*hap5*	CHGG_03369	− 1.6	3.19E−19	Down
	Clr1	*clr1*	CHGG_04631	− 3.1	4.29E−58	Down
	Clr2	*clr2*	CHGG_04595	− 2.3	3.25E−09	Down
Photoreceptor and light related regulators	LaeA	*LaeA*	CHGG_01690	− 1.7	0.000133	Down
	VeA	*VeA*	CHGG_10370	− 0.5	2.25E−13	Down
	VelB	*VelB*	CHGG_09077	− 1.7	1.37E−42	Down
	ENV1	*env1*	CHGG_00392	1.5	1.05E−12	Up

**Fig. 2:**
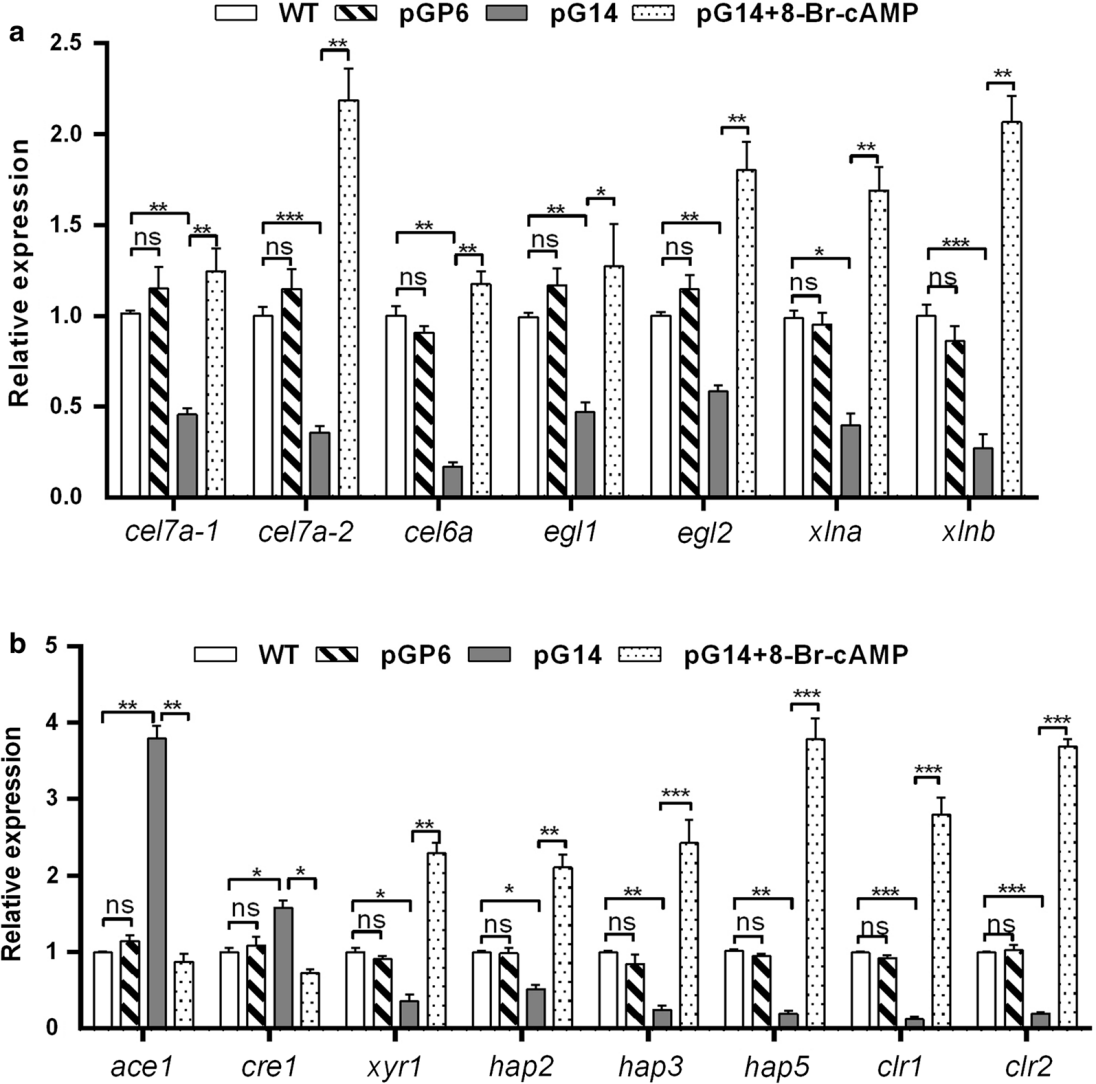
Knockdown of *gna1* results in differential expression of cellulase and xylanase genes as well as their transcriptional regulators. **a** Transcript levels of cellulase and xylanase genes were detected by qRT-PCR in the wild-type strain, pG14 mutant, pGP6 mutant, and pG14 mutant with the addition of 2 mM 8-Br-cAMP. **b** Transcript levels of transcriptional regulators were detected by qRT-PCR in the wild-type strain, pG14 mutant, pGP6 mutant, and pG14 mutant with the addition of 2 mM 8-Br-cAMP. The transcripts all were normalized against β-actin amplified with primers qActin (s) and qActin (as) (Additional file 1: Table S1). There was a significant difference between the mutant and wild-type strain, as indicated by an asterisk (P-value < 0.05 in t-test analysis) or by two asterisks (P-value < 0.01 in t-test analysis). Experiments were performed in triplicate

We further carried out qRT-PCR verification of the RNA-Seq results regarding characterized cellulase and xylanase genes as well as the TFs, and the results were highly consistent with the DEG data (Fig. 2a, b and Table 1). The transcription levels of all cellulase and xylanase genes dropped dramatically in pG14 (approximately two- to sixfold), yet they recovered in pGP6 almost to the wild-type level, particularly regarding the *cel7a*-*2*, *cel6a*, and *xlnb* genes (Fig. 2a). The results indicated an interaction between the G protein pathway and cellulase and xylanase synthesis. Regarding cellulase expression, the heterotrimeric G protein-activated cAMP/PKA signaling transduction pathway has been well studied in a number of model fungi [42, 43]. However, the pathway may function differently in different fungi. In *Penicillium decumbens*, the *cel7A*-*2* transcription increased in the Δ*pga3* strain (*pga3* encodes a group III G protein α subunit), while cellulase activity in the medium was not affected [29]. Instead, in *T. reesei*, both Gna1 and Gna3 play significant roles in the regulation of cellulase expression and positive correlation was observed between intracellular cAMP concentration and cellulase expression levels [42, 43]. Additionally, in *A. nidulans*, deletion of *pkaA* resulted in increased hydrolytic enzyme secretion [7]. Our results in *C. globosum* are similar to that in *T. reesei* and *A. nidulans.*

It has been reported that the most important role of cAMP is to activate cAMP-dependent PKA, which in turn initiates a phosphorylation cascade and activates/inactivates further target genes [28]. Most of the cellulase genes are regulated in a consistent manner, suggesting fine-tuned cooperation between the respective TFs. It has been found that, in *P. decumbens*, deletion of *pga3* resulted in impaired amylase production, and significantly decreased transcription of the major amylase gene *amy15A* [29]. In this study, the qRT-PCR results showed the transcription levels of *ace1* and *cre1* were dramatically increased in pG14 (3.8- and 1.6-fold, respectively), yet the levels decreased in pGP6 nearly to the wild-type levels. As expected, the transcription of *xlnR*, *clr1/2* and the *hap2/3/5* complex showed converse results (Fig. 2b). Of all the detected regulators, *ace1*, *clr1/2* and the *hap2/3/5* complex showed the highest variation (4- to eightfold), as they may act as the major regulators of cellulase production. In conclusion, the RNA-Seq and qRT-PCR results clearly demonstrated that the group I Gα protein encoded by *gna1* has a positive effect on cellulase and xylanase gene expression by regulating TFs in *C. globosum*.

### Secondary messenger cAMP affects cellulase and xylanase gene transcription and is positively regulated by Gα protein and negatively regulated by PKAR

One of the targets of the heterotrimeric G protein is the enzyme adenylyl cyclase that converts ATP to cAMP for use as a secondary messenger in filamentous fungi, as in other eukaryotic organisms [24–27]. Previous work revealed that intracellular levels of cAMP were modulated in a Gna1-dependent manner in *C. globosum* [34]. The cAMP level in the pG14 mutant was clearly reduced relative to that in the wild-type strain, while pGP6 increased cAMP accumulation nearly to the wild-type level [34]. Interestingly, in the wild-type strain, a dual effect of 8-Br-cAMP was observed on the expression of *CgcheA*, which is responsible for ChA biosynthesis. 8-Br-cAMP of low concentration (below ~ 2 mM) could stimulate transcription of *CgcheA*, but high concentration (above ~ 2 mM) repressed *CgcheA* expression [34].

To test whether diminished expression of the Gα subunit in the pG14 mutant affects cAMP synthesis in vivo, leading to the regulation of cellulase production in the mutant, we utilized an analog of cAMP (8-Br-cAMP) that has a similar function to cAMP in culture. Interestingly, when 2 mM 8-Br-cAMP was added to the broth, the cellulose-utilizing ability of the pG14 mutant was clearly stimulated. For example, the diameter of the zone of clearance on CMC agar was enlarged by 3.1-fold (Fig. 1a). The cellulase and xylanase activity increased 4.8- and 4.4-fold, respectively (Fig. 1d, f). We further used qRT-PCR to determine whether this was due to activation of cellulase and xylanase gene expression in the pG14 mutant. We found that 2 mM 8-Br-cAMP restored the expression of cellulase and xylanase genes in pG14, and some expression levels even exceed the wild-type level (approximately 1.2- to 2.2-fold compared to the wild-type strain). For instance, the transcription level of the *cel7a*-*2* and *xlnb* genes compared to the levels in pG14 increased strikingly by 6.1- and 7.7-fold, respectively (Fig. 2a). To determine whether the cAMP level affecting the transcription regulators led to the activation of expression of the cellulase and xylanase genes, we also detected the expression of TFs. The qRT-PCR results revealed that the addition of 8-Br-cAMP could promote the transcription of *clr1/2*, the *hap2/3/5* complex and *xyr1* while inhibiting the transcription of *ace1* and *cre1* (Fig. 2b). These findings, therefore, demonstrated that cAMP is positively regulated by Gα protein and has an important role in cellulase and xylanase gene transcription.

### Positive effect of Gna1 on expression of the *cel7a*-*2* gene and cellulase activity according to different carbon sources

Previous studies have suggested that cellobiohydrolase gene expression and the cellulolytic activity profile vary depending on the carbon source used in induction experiments in fungi [10, 19, 44]. But the regulatory mechanism acts differently in different fungi. For example, in *P. decumbens*, the regulatory effects of PGA3 are carbon source-independent. The G protein-cAMP signaling pathway transduces various carbon source signals and regulates the expression levels of specific TF genes (mainly *amyR*), followed by influencing the expression of amylases and cellulases in opposite directions [31]. In *T. reesei*, cAMP regulates the expression of cellulase in a carbon source-dependent manner. The expression of *cel7a* and *cel6a* genes was higher in the presence of sophorose than in the presence of other carbon sources [8]. However, little is known about the nature of the inducer, or the signaling pathways controlling cellulase gene expression in *C. globosum*. To demonstrate whether this process is regulated by the Gna1-mediated G protein-cAMP signaling pathway, the expression of the *cel7a*-*2* gene and cellulase activity were analyzed after growing pG14, pGP6, and the wild-type strain in MCC medium and adding different carbon sources.

**Fig. 3:**
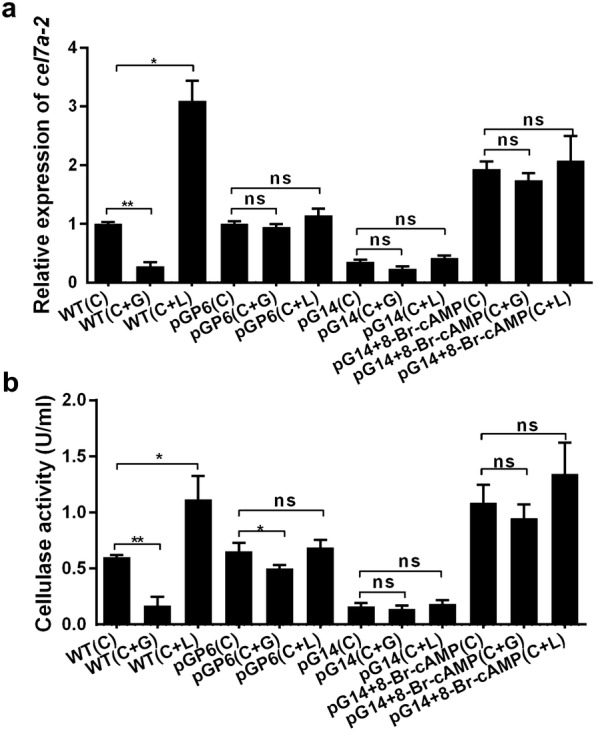
Effect of *gna1* on expression of the cellobiohydrolase *cel7a*-*2* and cellulase activity under conditions involving different carbon sources. **a** Transcript levels of *cel7a*-*2* genes were detected by qRT-PCR in different strains using the primers qCel7a-2 (s) and qCel7a-2 (as). All the transcripts were normalized against β-actin amplified with primers qActin (s) and qActin (as) (Additional file 1: Table S1). **b** Cellulase activity in different strains. Mycelia were grown in 200 ml MCC medium (C) or MCC medium supplemented with 1% glucose (G) or 1% lactose (L). After 8 days of incubation, the fermentation broth was used as the enzyme source. The statistical significance is indicated by an asterisk (P-value < 0.05 in t-test analysis) or by two asterisks (P-value < 0.01 in t-test analysis). Experiments were carried out in biological triplicates

As shown in Fig. 3a, in the wild-type strain, *cel7a*-*2* expression increased significantly (3.1-fold) when the fungus was grown in MCC medium with lactose. When the wild-type strain was grown in MCC medium with glucose, *cel7a*-*2* expression was sharply repressed (0.3-fold). The results showed that, in the wild-type strain, carbon catabolite repression was active regarding the expression of cellulases. Lactose is an inducer while glucose is a repressor. However, in contrast to what was observed in the wild-type strain, the expression of *cel7a*-*2* in pG14 and pGP6 did not change significantly in the presence of either lactose or glucose. Even when 2 mM 8-Br-cAMP was added to the broth, the changes of *cel7a*-*2* expression seemed to be not obvious (Fig. 3a). Similar results were observed in the cellulase activity assays (Fig. 3b). These results indicated that the transduction of carbon source signals is dependent on *gna1*. It is the Gna1-mediated G protein-cAMP signaling pathway that transduces various endogenous carbon source signals into cells and regulates the expression of cellulase genes. Seibel et al. [23] found that constitutive activation of Gna1 does not overcome inducer dependence of cellulase formation in *H. jecorina*, and declared that Gna1 does not transmit the essential inducing signal. With our findings, we concluded that although G protein-cAMP signaling pathway is crucial for transmitting signals and regulating cellulase genes transcription, the signal of inducers are required for activation of the process as well.

### G protein-cAMP signaling pathway regulating cellulase production in *C. globosum* involved in the light signaling mechanism

Besides the response to different substrates, cellulase gene transcript levels are also modulated by environmental factors especially light and by ENVOY (ENV1), the central component of the light signaling machinery in *T. reesei* [41]. The photoreceptor ENV1 mainly established connections with the heterotrimeric G-protein pathway and triggers posttranscriptional regulation of cellulase expression with light response. In addition, the Velvet complex, composed of mainly LaeA, VeA and VelB, coordinate the light signal with fungal development and secondary metabolism in *A. nidulans* and regulate cellulase gene expression in *T. reesei* [45]. In *T. reesei*, the VeA homologue VEL1 serves as a molecular link between light signaling, development and secondary metabolism [46].

**Fig. 4:**
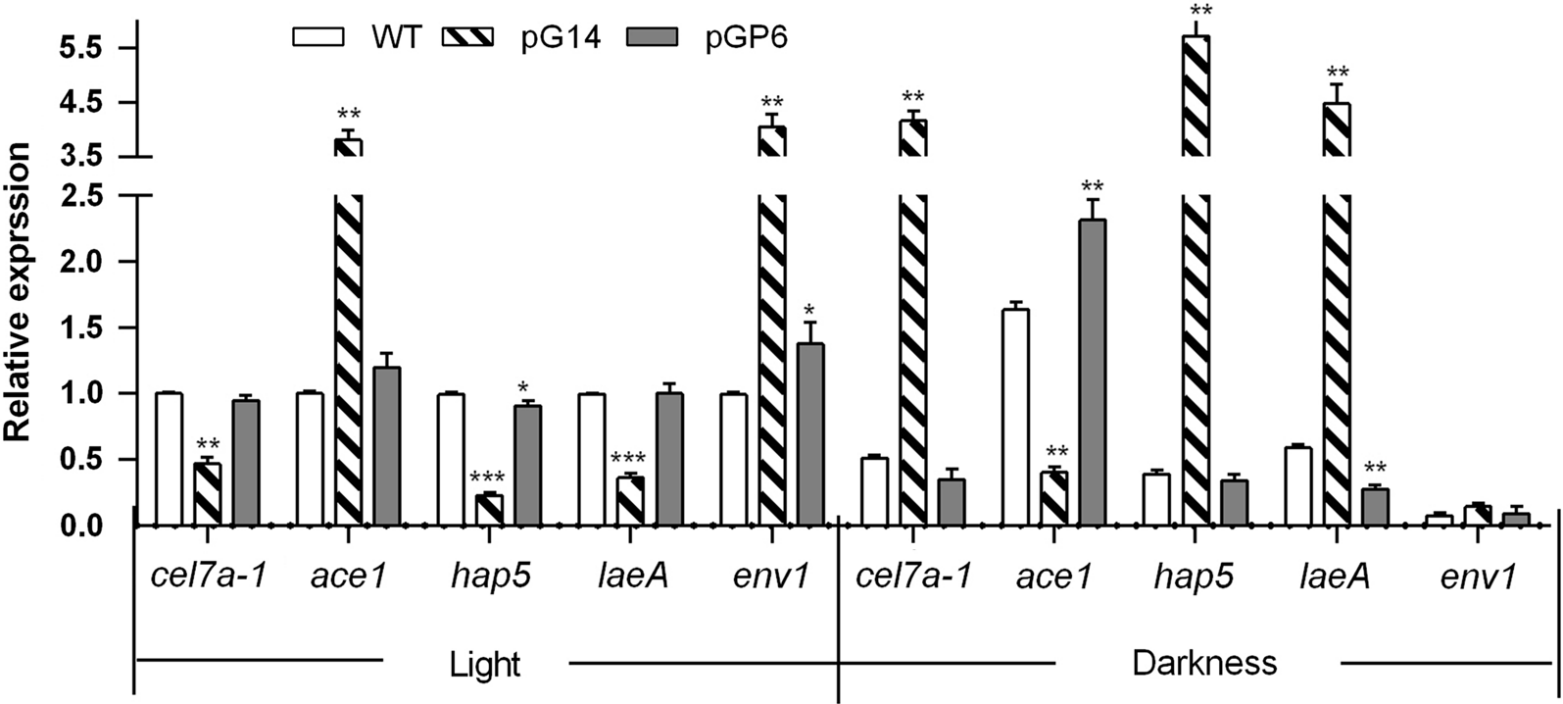
Effect of *gna1* on expression of the cellulase genes and transcription factors and regulators in light and darkness conditions. The strains were grown in MCC medium under constant light (2000 lx) or darkness for 8 days at 28 °C. Transcript levels of *cel7a*-*2*, *ace1*, *hap5*, *laeA and env1* genes were detected by qRT-PCR in different strains. All the transcripts were normalized against β-actin amplified with primers qActin (s) and qActin (as) (Additional file 1: Table S1)

We wondered whether G protein-cAMP signaling pathway regulating cellulase production in *C. globosum* through transmitting nutrient signals involved in the light signaling mechanism. Hence, the expression of cellulase genes and related regulators were analyzed in light and darkness. The qRT-PCR results showed that the function Gna1 impacts cellulase gene expression is dependent on light. When cultured in light, knockdown of Gna1 downregulated the expression of *hap5* and *laeA* and led to a strong decrease of cellulase transcripts (*cel7a*-*2* gene) upon growth on cellulose (Fig. 4). The *laeA* expression decreased to 36.3% compared to the wild type, which indicated that VelB/VeA/LaeA complex may coordinates light signal with cellulase production (Fig. 4). Interestingly, the photoreceptor *env1* transcripts increased more than fourfolds in pG14 while decreased nearly to the normal lever in pGP6, which indicated that Gna1 negatively regulates *env1* transcript levels in light (Fig. 4). However, in contrast to what was observed in light, decreased *cel7a*-*2* transcript level was observed in the wild type in darkness. This means that light is required for the expression of cellulase genes. Unexpectedly, knockdown of *gna1* upregulated the expression of *hap5* and *laeA*, while downregulated the expression of *ace1*, resulting in more than fourfolds increase of the *cel7a*-*2* transcript level (Fig. 4). The transcript levels of *env1* were hardly detected in all the mutants and the wild type without light (Fig. 4). The results indicated that ENV1 also impacts cellulase gene regulation dependent on light and acts at least in part via modulation of the cAMP pathway. These findings, therefore, demonstrated that G protein-cAMP signaling pathway regulating cellulase production involved in the light signaling mechanism, which is similar to the results in *T. reesei* [41, 45]. Furthermore, we hypothesize that VelB/VeA/LaeA complex and ENVOY probably work as downstream effectors that dictate expression of the cellulase genes by interacting with G protein/cAMP/PKA signaling. While, the specific mechanism of this regulatory network correlated with light conditions needs further study.

## Conclusion

**Fig. 5:**
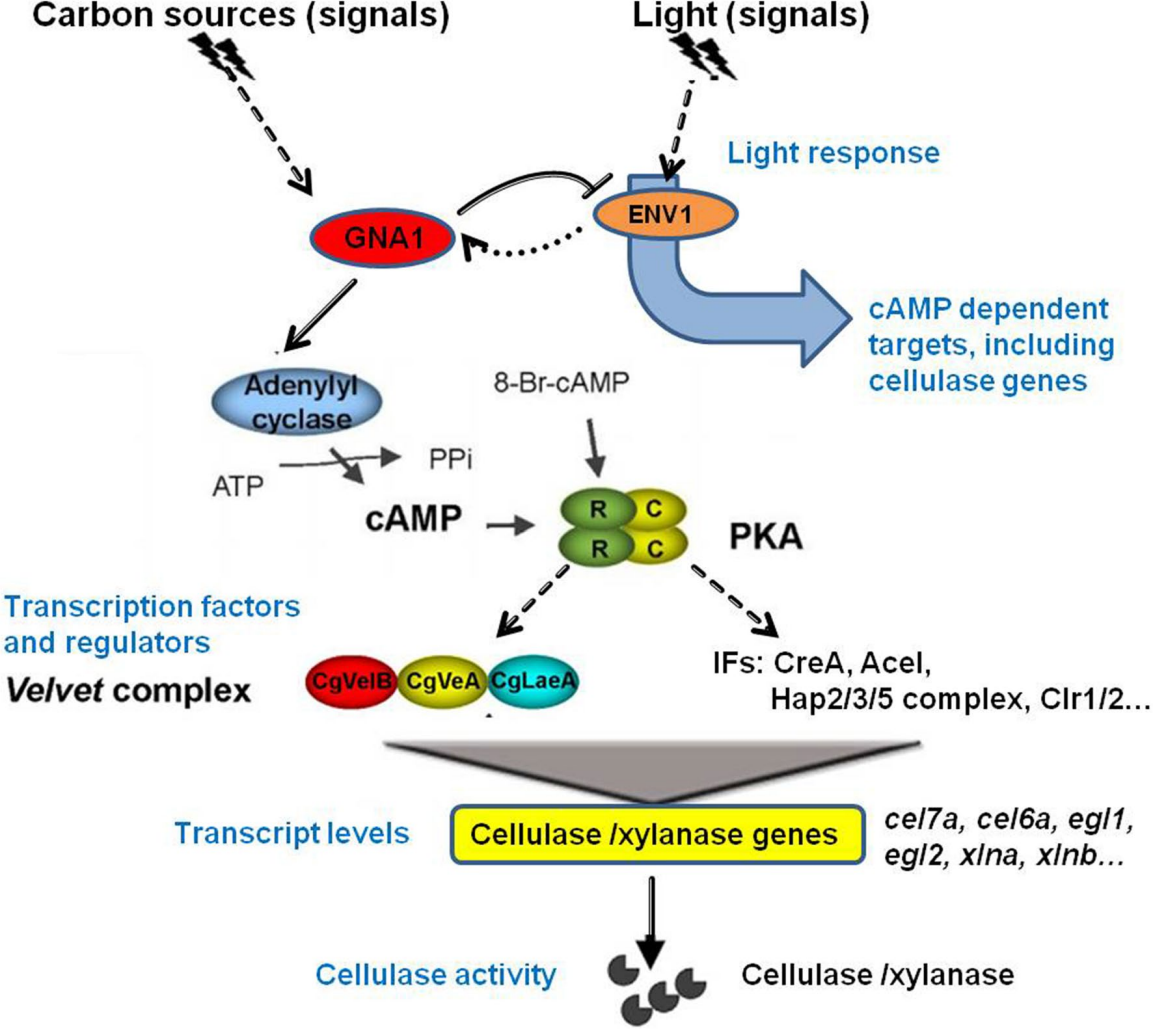
Schematic diagram of the role of the Gα-cAMP/PKA signaling pathway in regulating the production of extracellular-degrading enzymes in *C. globosum*. Solid lines represent possible direct regulations while dashed lines represent indirect regulations

The present study contributes to a better understanding of the important role of the G protein-cAMP signaling pathway in the regulation of cellulase expression in *C. globosum* (Fig. 5). This pathway is a prime candidate for sensing and transmission of the extracellular signals, including carbon sources and light. Signals are transmitted to the TFs or other downstream effectors such as VelB/VeA/LaeA complex and ENV1, resulting to the regulation of cellulase transcription. This is the first report discussing a potential role for Gna1 in the regulation of cellulase secretion and the transcriptional regulation mechanism in *C. globosum.* There are a large amount of cellulases and hemicellulases in the genome of *C. globosum* CBS148.51 by Carbohydrate-Active EnZymes (CAZy) database (unpublished data). Therefore, unraveling signal transduction in this fungus will help with understanding the regulatory networks, further with facilitating the metabolic engineering to improve the yield of cellulase and seeking practical application of reuse of cellulolytic wastes.

## Additional file

**Additional file 1: Table S1.** Primers used in this study. **Table S2.** Comparison of cellulase activity/gene expression of *C*. *globosum* NK102 cultured for 4 days and 8 days in MCC medium. **Table S3.** Biological processes indicated by the Gene Ontology (GO) analysis of differentially expressed genes (DEGs) in the *gna1*-silenced mutant pG14. **Figure S1.** Differentially expressed genes (DEGs) in the starch and sucrose metabolism pathway according to RNA-Seq results.

## Abbreviations

EG: endoglucanase
CBH: cellobiohydrolase
BG: β-glucosidase
GH: glycohydrolases
cAMP: cyclic adenosine monophosphate
CAZy: carbohydrate-active enzymes
CMC: carboxymethyl cellulose
PKA: cAMP-dependent protein kinase catalytic subunit A
β-PNG: β-nitro phenyl glucopyranoside
GPCR: G-protein coupled receptor
cAMP: cyclic adenosine monophosphate
PKAR: regulatory subunits of cAMP-dependent protein kinase A
RNAi: RNA interference
DEGs: differentially expressed genes
qRT-PCR: quantitative reverse transcription PCR
FPase: filter-paper-hydrolysing
