# Supplementary material for: Regulation of the Gα-cAMP/PKA signaling pathway in cellulose utilization of Chaetomium globosum
Source: Microb Cell Fact. 2018 Oct 11;17:160. doi: 10.1186/s12934-018-1008-6 (PMC6182798; doi:10.1186/s12934-018-1008-6)
Supplement: Supplementary file 1 — Additional file 1: Table S1. Primers used in this study. Table S2. Comparison of cellulase activity/gene expression of C. globosum NK102 cultured for 4 days and 8 days in MCC medium. Table S3. Biological processes indicated by the Gene Ontology (GO) analysis of differentially expressed genes (DEGs) in the gna1-silenced mutant pG14. Figure S1. Differentially expressed genes (DEGs) in the starch and sucrose metabolism pathway according to RNA-Seq results. [file 12934_2018_1008_MOESM1_ESM.docx]

**Additional data for the manuscript submitted to Microbial Cell Factories**

**Regulation of the Gα-cAMP/PKA Signaling Pathway in Cellulose Utilization of *Chaetomium globosum***

Yang Hu^1^, Yanjie Liu^2^, Xiaoran Hao^2^, Dan Wang^3^, Oren Akhberdi^3^, Biyun Xiang^2^, Xudong Zhu^2,3,^*

^1^ Department of Pathogen Biology, School of Basic Medical Sciences, Tianjin Medical University, Tianjin, China.

^2^ Beijing Key Laboratory of Genetic Engineering Drug and Biotechnology, Institute of Biochemistry and Biotechnology, School of Life Sciences, Beijing Normal University, Beijing, China

^3^ National Key Program of Microbiology and Department of Microbiology, College of Life Sciences, Nankai University (DMNU), Tianjin, China.

*Corresponding author.

E-mail: [zhu11187@bnu.edu.cn](mailto:zhu11187@bnu.edu.cn). Tel: (+86) 010-58804266

Address: No. 19, XinJieKouWai St., HaiDian District, Beijing, 100875, China

**Table S1. Primers used in this study.**

| **Primer name** | **Sequence (5’-3’)** |
| --- | --- |
| qActin (s) | AACCGAGGCTCCCATCAAC |
| qActin (as) | TCACGGACGATTTCACGCTC |
| qCel7a-1 (s) | GGTGCAAGGCGAAGACCAACTAT |
| qCel7a-1 (as) | TGAGCGAAAGCATCTCGTAGCG |
| qCel7a-2 (s) | CGATGGTCTTGAAGTGGTTGTT |
| qCel7a-2 (as) | TTCACCCTCGTGACCCAGT |
| qCel6a (s) | CTTCTCGGGTGTTCAGATGTGGG |
| qCel6a (as) | TCACGATCCGGGAGATCGTAGAC |
| qEgl1 (s) | GACGCCTGAAGTCCATCCCAA |
| qEgl1 (as) | CTGCTGGTGTAGTCGGTGCCTT |
| qEgl2 (s) | TCCAGAAGGTCTTGAAGGCGT |
| qEgl2 (as) | TCCCGTCAACTTCGTCTATTCAG |
| qXlna (s) | CTCGGTCATGGAGACGCACATC |
| qXlna (as) | CGACGGCATAGTCGGTCTTGG |
| qXlnb (s) | ACCCGCTACAAGGGCAAGA |
| qXlnb (as) | GGACGCCAGAGGCAGAGTT |
| qAce1 (s) | ACTGGTCAGCCCATTGATAAGAT |
| qAce1 (as) | GTGCTTGTCGTTGTTGTGGC |
| qCre1 (s) | GCAGCAAACTGAAGCCGAGAT |
| qCre1(as) | TTGATGGTGTCCGCTCTTATTCC |
| qXyr1(s) | GGAGCGAGTCTGCGATGGTG |
| qXyr1(as) | CGTGGAAGTTGCCGTTCTGGT |
| qHap2 (s) | GTCCACGCAACATCTGTCGCA |
| qHap2 (as) | CCCGCCCTGGCATCCTTCTT |
| qHap3 (s) | ACGAGGAAGCACAAATGAAC |
| qHap3 (as) | GCTCCCGGTACTTGGAAAGGT |
| qHap5 (s) | GGCAAATGCCTGTTTACGACC |
| qHap5 (as) | GCCGAAATCATCTTGACCTCTGG |
| qClr1(s) | GTCAGCCAACTCATTTATTCCAAG |
| qClr1(as) | CAAAGAATCGTAGCCTCAACACC |
| qClr2(s) | CCAAGACAGCACCAAGACCAG |
| qClr2(as) | GGCCGTCAAACGAATGTAAAGA |
| qLaeA(s) | TCATAGTGCTCCCCTCCACA |
| qLaeA(as) | AGGTCAACGCCGACATGAAT |
| qEnv1(s) | CGGCTTTCAGATTGACTTGGT |
| qEnv1((as) | GCCTTGGGCCTGTATTGTTG |

**Table S2. Comparison of cellulase activity/gene expression of *C*. *globosum* NK102 cultured for 4 days and 8 days in MCC medium.**

| **Cellulase activity/gene expression** | **4 d** | **8 d** |
| --- | --- | --- |
| Cellulase activity(U/ml) | 0.40±0.03 | 0.63±0.06* |
| FPase activity(U/ml) | 0.15±0.01 | 0.24±0.05* |
| Relative *cel7a-1* gene expression | 1.01±0.01 | 2.57±0.21** |
| Relative *egl-1* gene expression | 1.00±0.01 | 3.33±0.31** |

*, P-value <0.05 in t-test analysis; **, P-value <0.01 in t-test analysis.

**Table S3. Biological processes indicated by the Gene Ontology (GO) analysis of differentially expressed genes (DEGs) in the *gna1*-silenced mutant pG14.**

| **Gene Ontology term** | **Number of differential genes** | | **Corrected P-value** |
| --- | --- | --- | --- |
| cellular process | | 847 | 0.00123 |
| proteolysis | | 31 | 0.00412 |
| cellular protein metabolic process | | 205 | 0.01903 |
| protein metabolic process | | 264 | 0.03555 |
| cellular metabolic process | | 709 | 0.06618 |
| protein localization | | 43 | 0.06884 |
| vesicle-mediated transport | | 19 | 0.07912 |
| cell cycle process | | 16 | 0.09556 |
| cellular macromolecule catabolic process | | 27 | 0.09817 |
| macromolecule localization | | 50 | 0.12908 |
| cell cycle phase | | 14 | 0.14324 |
| modification-dependent protein catabolic process | | 24 | 0.15464 |
| protein catabolic process | | 24 | 0.15464 |
| modification-dependent macromolecule catabolic process | | 24 | 0.15464 |
| cellular protein catabolic process | | 24 | 0.15464 |
| proteolysis involved in cellular protein catabolic process | | 24 | 0.15464 |
| cellular localization | | 26 | 0.17467 |
| cellular carbohydrate metabolic process | | 49 | 0.17469 |
| protein transport | | 41 | 0.17497 |
| establishment of protein localization | | 41 | 0.17497 |
| tRNA aminoacylation for protein translation | | 32 | 0.19922 |
| M phase | | 12 | 0.20324 |
| carboxylic acid metabolic process | | 110 | 0.23516 |
| oxoacid metabolic process | | 112 | 0.25695 |
| hexose metabolic process | | 25 | 0.30718 |
| mitotic cell cycle | | 13 | 0.30865 |
| cell cycle | | 17 | 0.31095 |
| organic acid metabolic process | | 112 | 0.31706 |
| amino acid activation | | 32 | 0.32779 |
| tRNA aminoacylation | | 32 | 0.32779 |
| cellular ketone metabolic process | | 113 | 0.36597 |
| sulfur compound metabolic process | | 20 | 0.4415 |


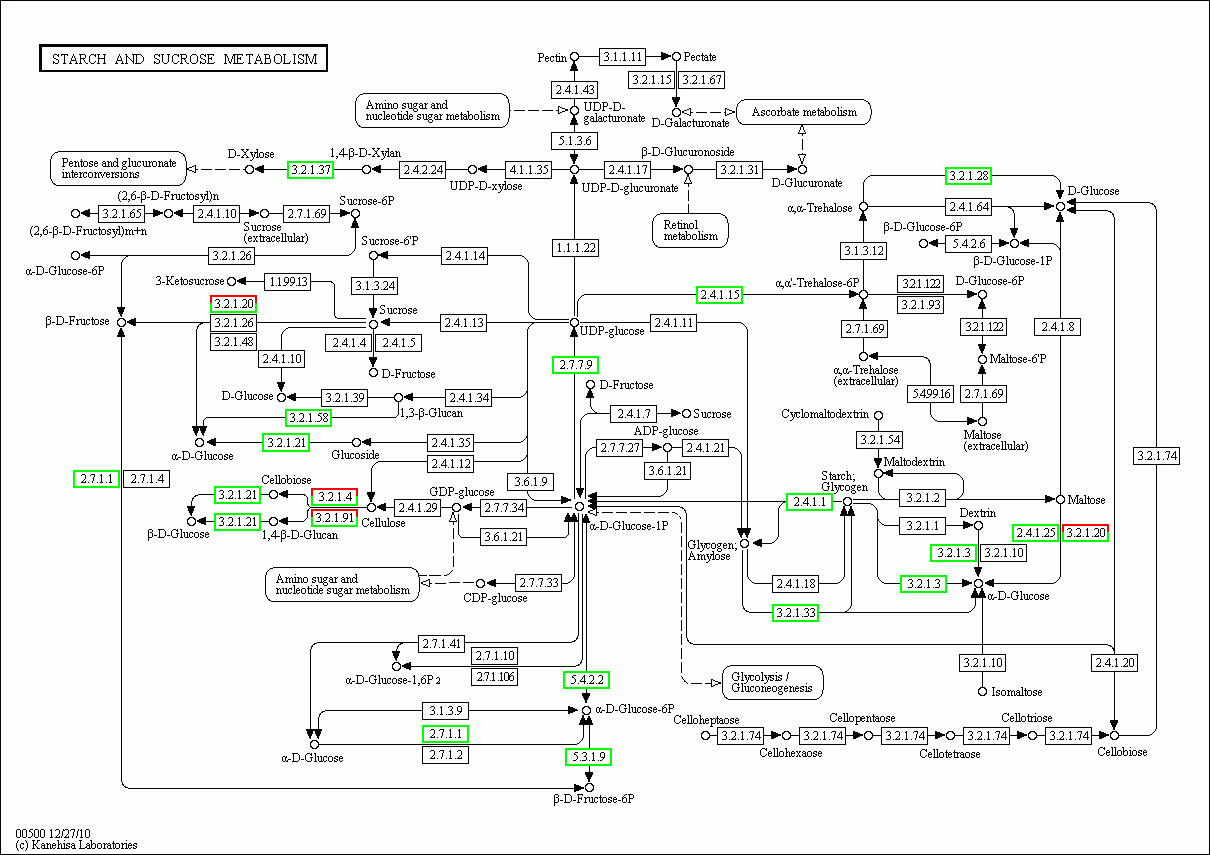


**Figure S1:** Differentially expressed genes (DEGs) in the starch and sucrose metabolism pathway according to RNA-Seq results. The green box means the gene expression decreased; the red box means the gene expression increased. P-values were used to evaluate the statistical significance of expression differences. A false discovery rate (FDR)-corrected P-value <0.001 and an absolute log_2_Ratio value≥1 were used to identify the DEGs and differentially expressed tags (DETs).
